# Antibody therapies for the prevention and treatment of viral infections

**DOI:** 10.1038/s41541-017-0019-3

**Published:** 2017-07-10

**Authors:** Georgina Salazar, Ningyan Zhang, Tong-Ming Fu, Zhiqiang An

**Affiliations:** 1 Texas Therapeutics Institute, Brown Foundation Institute of Molecular Medicine, The University of Texas Health Science Center at Houston, Houston, TX USA; 2 Merck Research Laboratories, Merck and Co., Inc., Kenilworth, NJ USA

## Abstract

Antibodies are an important component in host immune responses to viral pathogens. Because of their unique maturation process, antibodies can evolve to be highly specific to viral antigens. Physicians and researchers have been relying on such high specificity in their quest to understand host–viral interaction and viral pathogenesis mechanisms and to find potential cures for viral infection and disease. With more than 60 recombinant monoclonal antibodies developed for human use in the last 20 years, monoclonal antibodies are now considered a viable therapeutic modality for infectious disease targets, including newly emerging viral pathogens such as Ebola representing heightened public health concerns, as well as pathogens that have long been known, such as human cytomegalovirus. Here, we summarize some recent advances in identification and characterization of monoclonal antibodies suitable as drug candidates for clinical evaluation, and review some promising candidates in the development pipeline.

## Introduction

The earliest application of antibodies as a treatment for viral infections can be traced back to the early 20th century, use of sera from infected humans who had recovered from the same infection.^1, 2^ This crude treatment regimen, serum therapy, was gradually replaced by antibodies purified from pooled sera, intravenous immune globulin (IVIG).^3^ Despite the success of both serum therapy and IVIG, no significant progress was made in the generation of antibodies as therapies until the hybridoma method was developed, enabling isolation of monoclonal antibodies (mAbs) from immunized mice in 1975.^4^ Since the mid-1980s, several methods have been developed for the efficient isolation of mAbs against viruses from human and animal sources. One method involves using an antigen to pan antibody libraries constructed from immunoglobulin V_H_ and V_L_ variable regions genes of non-immune, vaccinated, or naturally infected individuals. In this method, antibody libraries are presented to antigens by display, for instance on phage,^5, 6^ bacteria,^7^ yeast,^8^ or mammalian cells.^9^ In other methods, antibodies are cloned from single-memory B cells^10–13^ or plasma B cells^14–16^ isolated from vaccinated or naturally infected animals and human donors. mAbs have also been generated from immune sera using an approach that combines proteomics and reverse genetics.^17, 18^ More recently, heavy and light chain paired mAbs have been generated by deep sequencing of the B-cell IgG repertoire.^19, 20^ This review focuses on approaches to generate therapeutic mAbs to fight viral infection, examples of mAb therapies for viral infections, and the challenges of developing such therapies.

### Strategies for generation of therapeutic antibodies for viral infections

#### Phage displayed antibody libraries

**Fig. 1:**
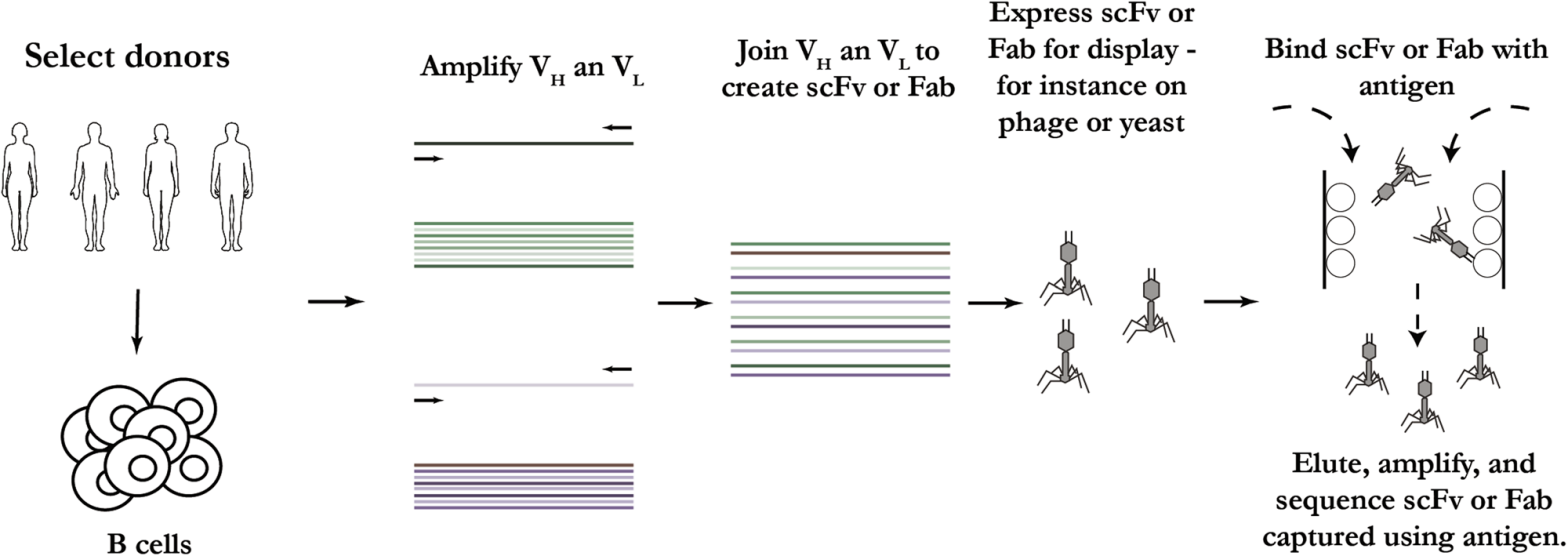
Schematic representation of generation of mAbs using phage display. This method has the advantage of being relatively easy with the potential to generate VH and VL combinations not found in nature. The diagram was generated based on a combination of publications^6, 21^

mAbs can be isolated from immunized or infected humans or animals using a library of displayed antibodies (Fig. 1). Genes encoding the antibody heavy and light chains from B cells of immunized or infected humans or animals are cloned as Fab or scFv fragments and displayed, for instance on filamentous phage. Virus-specific antibodies are isolated by panning the libraries against antigens. This approach has been used to isolate potent neutralizing antibodies from the B cells of rhesus macaques immunized with recombinant adenoviruses carrying a synthetic gene encoding hemagglutinin (HA) of the influenza virus^6^ and from human IgM^+^ memory B cells of recent seasonal influenza vaccines.^5^ In addition to panning libraries constructed from antibody repertoires following infection, panning of native antibody libraries has yielded potent neutralizing antibodies against viral infections. For example, a broadly neutralizing HIV antibody (D5) was isolated from panning a native antibody library.^21^ It might be difficult to identify highly neutralizing antibodies when panning against native phage libraries, because of the lack of antibody somatic hypermutation process. This disadvantage may be overcome by an in vitro affinity maturation step or by panning of libraries constructed from immunized or infected human or animal donors in which antibody somatic hypermutations took place against a given virus. While phage display is an efficient way to generate viral neutralizing antibodies from immunized, or infected, or non-immune humans or animals, the resulting antibodies do not necessarily represent the natural antibody repertoire, since the antibody fragments are generated from the random paring of IgG heavy and light variable regions.^22^ Further, since a predefined and well-characterized antigen is required to pan the library, the approach is not suitable to identify new neutralizing epitopes of viral pathogens.^22^

#### Single-memory B cells

**Fig. 2:**
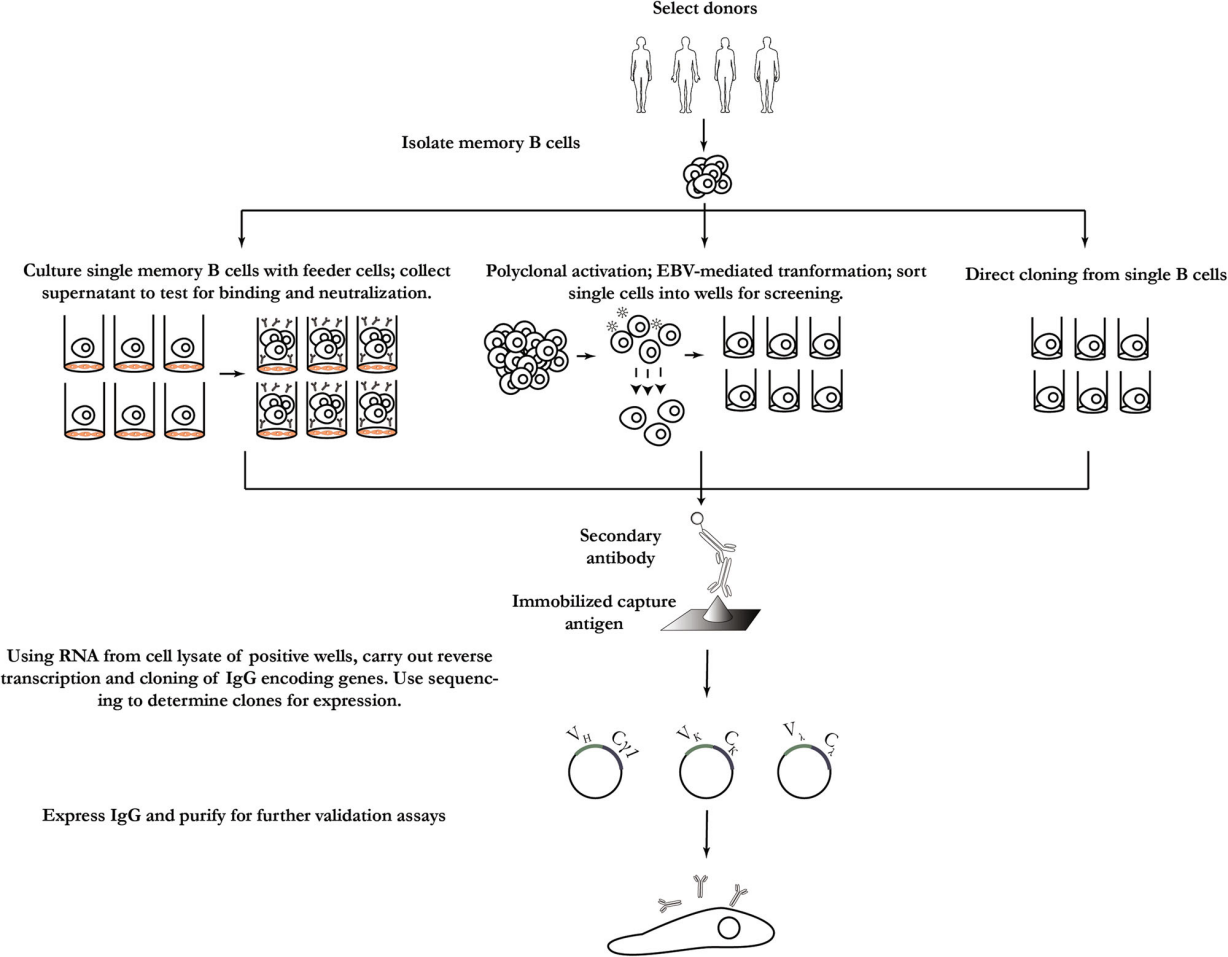
Schematic of isolation of mAbs from memory B cells. When these cells producing antibodies with desired characteristics can be found, a supply of these antibodies can be maintained by culture and cloning, immortalization, or direct cloning. The diagram was generated by summarizing multiple published papers. The diagram was generated based on a combination of publications^10–13, 22–27^

The ability to clone antibody encoding genes from single B cells of naturally infected or immunized individuals is a significant advance in isolating anti-viral mAbs. Three different approaches have been developed for the isolation of mAbs from single-memory B cells (Fig. 2). One approach involves isolating memory B cells specific for viral antigens by flow cytometry, followed by direct cloning of the antibody-encoding genes without culturing of the B cells. This approach has been applied to isolate neutralizing antibodies from naturally infected and immunized human donors for Zika,^23^ respiratory syncytial virus (RSV),^24^ HIV,^12^ and HPV.^25^ Antibodies were also isolated through this method from antigen-specific individual memory B cells of rhesus macaques immunized with gp140-F trimers based on the HIV-1 YU2 isolate.^26^

Since predefined antigens are required to identify the memory B cells then isolated by flow cytometry sorting, the above-mentioned approach is not suitable for identification of novel neutralizing targets. To identify novel target epitopes, first, a large number of memory B cells are cultured, and then, the culture supernatant is screened against various antigens. This approach has been used for the discovery of broad and potent neutralizing antibodies against HIV and the discovery of a new HIV-1 vaccine target.^11^ In this work,^11^ surface IgG-expressing memory B cells were enriched from peripheral blood mononuclear cells (PBMCs) of a HIV-1 infected donor by negative depletion with antibodies to CD3, CD14, CD16, IgM, IgA, IgD on magnetic beads. Individual memory B cells were then seeded in microtiterplates in the presence of feeder cells and conditioned media generated from mitogen-stimulated human T cells from healthy donors. The supernatants were collected 8 days later and screened for antigen-binding reactivity. In another protocol, the addition of cytokines interleukin (IL)-2, IL-21, and irradiated 3T3-msCD40L feeder cells can successfully stimulate memory B cells to produce high concentrations of IgG in the supernatant in 2 weeks.^10^

In a third approach, B cells are immortalized by Epstein-Barr Virus (EBV) in the presence of a toll-like receptor. This approach has been applied to the isolation of neutralizing antibodies against rabies, SARS-CoV, and other viruses.^13, 22, 27^

#### Single-antibody-secreting plasma B cells

**Fig. 3:**
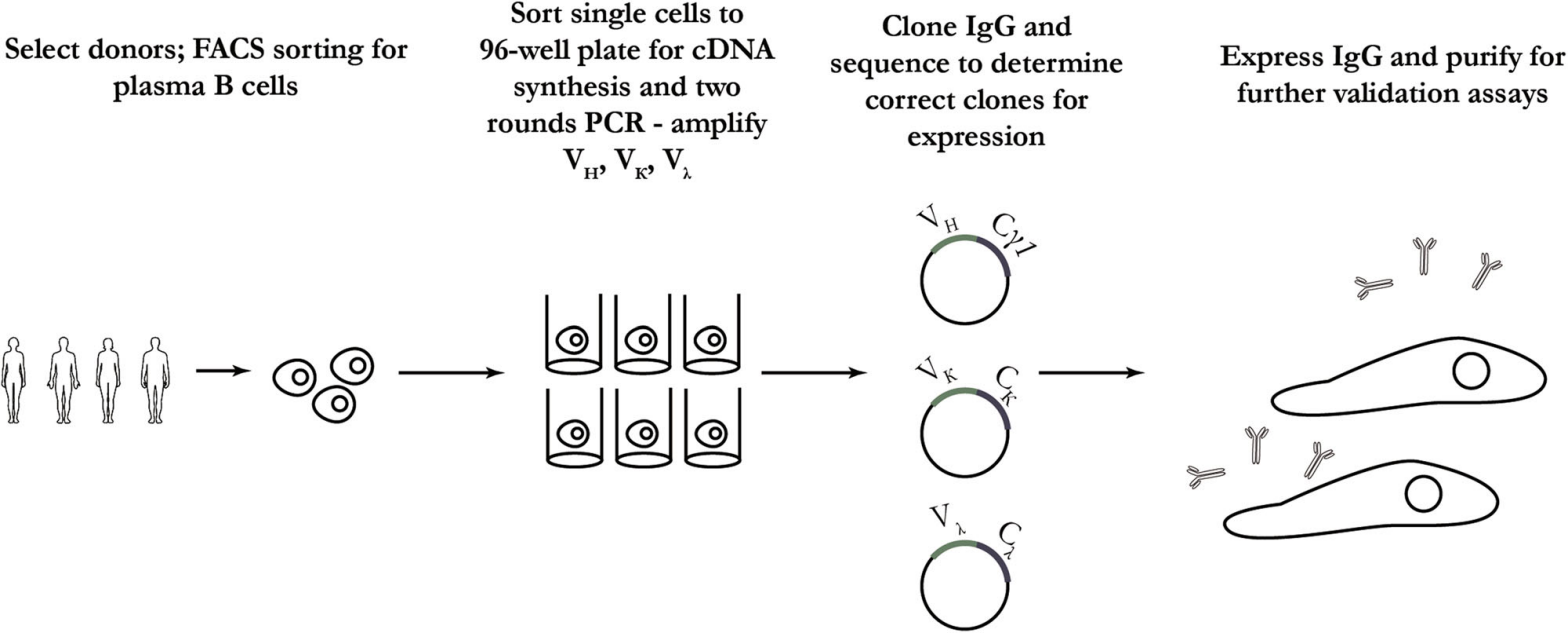
Overview of isolation, culture, and antibody gene cloning from plasma B cells. This method can be an efficient way of generating antibodies with desired specificity given the right antigen bait and timing. The diagram was generated based on a combination of publications^15, 16, 28–37^

It has been reported that antigen-specific plasma cells account for up to 40–90% of total IgG-secreting cells in peripheral blood one week after boost immunization.^28^ Methods for cloning IgG from human antibody-secreting cells have been developed (Fig. 3).^15, 29–32^ This approach has been applied for isolating neutralizing antibodies from naturally infected and immunized human donors for dengue^33^ and H1N1 influenza.^15, 34^ Protocols for cloning mAbs from nonhuman primate plasma and memory B cells have also been reported.^16, 35, 36^ In one report, a large panel of dengue targeting mAbs were cloned from single-antibody-secreting B cells of a rhesus macaque immunized with an experimental vaccine.^16^ Single-antibody-secreting B cells were identified and isolated by flow cytometry, using a panel of phenotype markers.^16^ To interrogate a large number of plasma B cells, a method was developed using microwell array chips. This method enables the analysis of live cells on a single-cell basis and offers rapid, efficient, and high-throughput (up to 234,000 individual cells) identification of antibody-secreting plasma cells.^37^

In addition to direct cloning of antibody encoding genes from plasma B cells, single plasma cells can also be cultured on a monolayer of immortalized stromal cells or with IL-6 stimulation; the cultured cells would be able to produce enough antibody for screening assays of parallel viral binding and neutralization for identifying rare antibodies.^29^

#### Proteomics-directed cloning of mAbs from serum

**Fig. 4:**
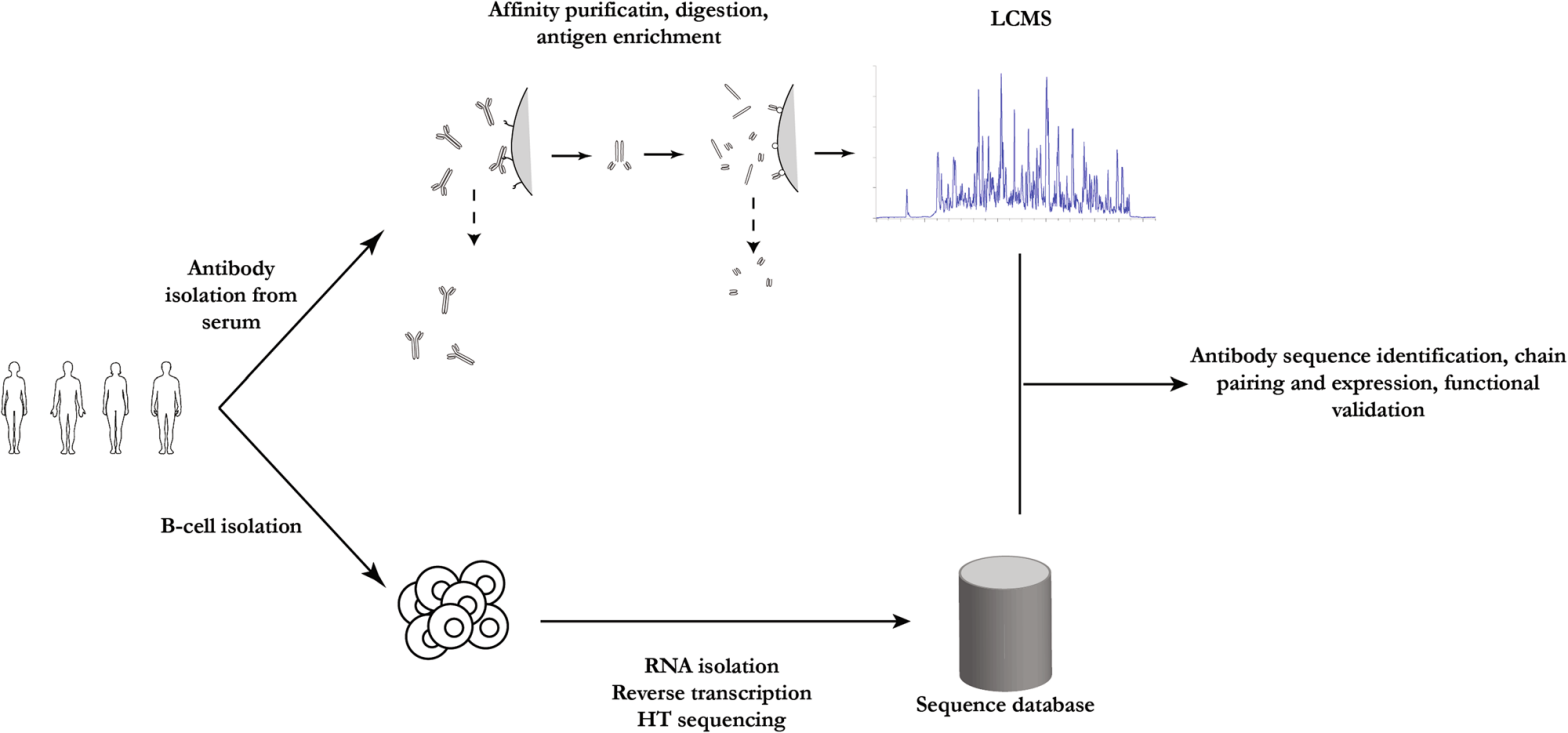
Representation of the combination of proteomics and high-throughput sequencing approaches to isolation of relevant mAbs from human donors. Analysis of nucleic acids from B cells combined with proteomic analysis of antibodies in serum provides a deeper understanding of the humoral response to viral infection and vaccinations. The diagram was generated based on a combination of publications^17, 18, 38, 39^

Antibodies cloned from single memory or plasma B cells represent the genetic antibody repertoire of an individual at a given time in response to a specific viral infection. However, this repertoire does not necessarily correspond to that of the antibodies present in circulation. Protocols have been developed to identify antigen-specific antibody sequences directly from circulating polyclonal antibodies in the sera of immunized or naturally infected animals or humans (Fig. 4). These protocols combine the power of proteomics and next-generation sequencing (NGS).^17, 18, 38, 39^ The approach involves affinity purification of antibodies with antigen specificity followed by analysis of proteolytically digested antibody fractions by liquid chromatography–mass spectrometry. Peptide spectral matches of antibody variable regions are obtained by searching a reference database created by NGS of the B-cell immunoglobulin repertoire of the immunized animal or human. Finally, heavy and light chain sequences are paired and expressed as recombinant mAbs.^17, 18, 38^ Using this approach, mAbs targeting HBV and human cytomegalovirus (HCMV) were cloned from human donors.^38^ This approach provides an alternative method to isolate potent viral neutralizing antibodies for therapeutic purposes. Further, it promotes a deeper understanding of the humoral response.

#### Deep sequencing of paired antibodies encoding genes from B cells

While cloning mAbs from individual memory or plasma B cells is a robust and efficient approach to isolate viral-specific antibodies, the approach provides only a snapshot and sample of the antibody genetic repertoire of an individual at a given time against a particular virus. Various deep-sequencing approaches can provide an unbiased picture of the B-cell repertoire based on either its heavy chain or light chain information, but the endogenous pairing of heavy and light chain is lost after bulk lysis of B-cell populations.^40^ Phylogenetic matching of heavy and light chains from antibody deep-sequencing data can provide an approximation of natural heavy and light chain pairing.^41^ High-throughput sequencing of paired heavy and light chain variable regions at the single-cell level has proven technically challenging. A method that allows the determination of a paired heavy and light chain variable region repertoire from millions of cells with high precision was recently reported.^19, 20, 42^ Briefly, flow-focusing is used to encapsulate single cells in emulsions containing magnetic beads for messenger RNA (mRNA) capture. The mRNA transcripts are then reverse-transcribed, physically linked to their partners by overlap extension PCR, and interrogated by high-throughput paired-end sequencing.^19^ Once adapted widely by the research community, this method will provide powerful information on antibody response to infection and vaccination, vaccine efficacy, and the cloning of potent viral neutralizing antibodies.^42^

### mAbs targeting viral infections

Table 1 shows mAbs in clinical development for the prevention and treatment of infectious viral diseases. These mAbs have been isolated from a variety of non-immune and immune sources, human and animal donors, using the range of strategies for generation of therapeutic antibodies introduced above.

**Table 1:** Antibody therapies in clinical trials for the prevention and treatment of viral infections

Virus	MAb, sponsor	Notes	Reference
HCMV	**CSJ148 (LJP538 and LJP539)**, Novartis	**CSJ148** is a combination of two anti-HCMV human mAbs that bind to and inhibit the function of viral HCMV gB (LJP538) and pentameric gH complex (LJP539). The two antibodies were isolated from EBV immortalized B cells. CSJ148 is in Phase 2 clinical trials (NCT02268526)	52
	**RG7667 (MCMV5322A and MCMV3068A)**, Genentech	**RG7667** is a combination of two mAbs that bind to glycoprotein complexes gH/gL (MCMV5322A) and pentameric gH complex (MCMV3068A). **MCMV5322A** is a human immunoglobulin antibody (IgG1κ) and is an affinity-matured version of MSL-109. **MCMV3068A** was isolated from an hybridoma screen in mice and subsequently humanized with an IgG1λ framework. Phase 2 clinical trials were completed for RG7667 (NCT01753167)	49
	**TCN-202**, Theraclone Sciences	**TCN-202** is a human mAb that targets AD-2, a linear, conserved, poorly immunogenic epitope on the N-terminal domain of HCMV gB. This mAb effectively neutralized infection and was observed to be well tolerated and non-immunogenic in initial clinical trials. However, development was discontinued after a Phase 1 adverse event (NCT01594437)	51
	**MSL-109/Serivumab**, NCRR, NIAID, Facet Biotech, Johns Hopkings Bloomberg School of Public Health, Sandoz Inc.	**MSL-109** is a human monoclonal IgG isolated from a HCMV seropositive individual that recognizes the viral glycoprotein H (gH). A Phase 2/3 trial of MSL 109 was completed (NCT00000836)	57
Influenza	**MHAA4549A (earlier known as 39.29)**, Genentech, Inc.	**MHAA4549A** is a human immunoglobulin G1 (IgG1) mAb that binds to a highly conserved epitope on the stalk of Group 1 and Group 2 influenza A hemagglutinins and blocks the hemagglutinin-mediated membrane fusion in the endosome, neutralizing all known human influenza A strains. MHAA4549A was cloned from a single human plasmablast cell isolated from an influenza vaccinated donor MHAA4549A is in Phase 2 clinical trials (NCT02623322)	62
	**VIS410**, Visterra, Inc.	**VIS410** targets a conserved epitope in the stem of influenza A hemagglutinin (HA). It was engineered using structural information on antibody–antigen interfaces. VIS410 is in Phase 2 clinical trials (NCT02989194)	151
	**CR6261**, Crucell Holland BV and the National Institute of Allergy and Infectious Diseases (NIAID)	Isolated from a healthy, vaccinated individual using phage display selection on recombinant H5 HA, mAb **CR6261** targets a highly conserved helical region in the membrane-proximal stem of HA1/HA2 from 1918 H1N1 influenza and H5N1 influenza. It uses the Ig VH1–69 germline segment. CR6261 is in Phase 2 clinical trials (NCT02371668)	70
	**CR8020**, Crucell Holland BV and Retroscreen Virology Ltd.	**CR8020** is a broadly neutralizing influenza hemagglutinin stem-specific human mAb. CR8020 was isolated from a B cell of a donor vaccinated against influenza. A Phase 2 clinical trial was completed for CR8020 (NCT01938352)	74
	**TCN-032**, Theraclone Sciences	One of a panel of mAbs derived from memory B cells of healthy human subjects, **TCN-032** targets an epitope in the ectodomain of the influenza matrix 2 protein M2e. This epitope, first identified with the isolation of the panel of mAbs including **TCN-032**, is highly conserved in influenza A viruses. A Phase 2 clinical study was initiated for TCN-032 (NCT01719874)	76
HIV	**VRC01**, NIAID	**VRC01** is a broadly neutralizing antibody targeting the CD4-binding site of HIV gp120. It was isolated from memory B cells of infected individuals using a targeted flow cytometry-based approach. VRC01 is in multiple Phase 2 clinical trials (NCT02664415, NCT02568215, NCT02716675)	40
	**3BNC117**, Rockefeller University, Weill Medical College of Cornell University, Brigham and Women’s Hospital, and the University of Cologne	3BNC117 is a broad and potent neutralizing antibody against the CD4-binding site of the HIV-1 Env protein. 3BNC117 is being tested in multiple Phase 2 clinical trials (NCT02446847, NCT02588586. NCT02850016)	92
	**10-1074**, Rockefeller University and the University of Cologne	Several antibodies including **10-1074** were isolated from B cells from a clade A-infected African donor using YU-2 gp140 trimers as bait. 10-1074 is a broadly neutralizing antibody (bnAb) that targets the V3-glycan supersite of HIV gp120. 10-1074 is currently in multiple Phase 1 clinical trials in combination with 3BNC117 (NCT02825797, NCT02511990, NCT02825797)	152
	**4E10, 2F5, 2G12**, Rockefeller University	**4E10, 2F5, 2G12** are broadly and potently neutralizing mAb specific for gp41. A Phase 1/2 clinical trial was completed for well-suppressed HAART-treated individuals during acute and early HIV-1 infection (NCT00219986)	153
	**PRO 140**, Amarex Clinical Research, CytoDyn, Inc., National Institute on Drug Abuse (NIDA), and Drexel University	**PRO 140** is an antibody used to treat HIV targeting host CCR5 receptors. Pro 140 is in multiple Phase 2/3 trials (NCT02859961, NCT02355184, NCT02483078,NCT02990858, NCT02438345, NCT02737306, NCT01272258)	99
	**Ibalizumab (TNX-355)**, Genentech	This anti-CD4 mAb is in Phase 3 trials (NCT02707861)	154
RSV	**Synagis (Palivizumab; MEDI-493)**, MedImmune	Approved for prophylaxis in infants at high risk for RSV, Synagis is a humanized mAb of IgG1 isoform. It targets RSV glycoprotein F	103
	**(Numax, MEDI-524)**, MedImmune	**Motavizumab** has completed Phase 3 trials for prophylaxis in infants at high risk for RSV. It is an affinity matured version of Palivizumab (NCT00129766)	106
	**Motavizumab—YTE (MEDI-557)**, MedImmune	**Motavizumab—YTE** is an Fc-modified, half-life extended derivative of motavizumab, with amino-acid substitutions M252Y/S254T/T256E. This mAb has completed Phase 1 trials (NCT01562938, NCT01475305, NCT00578682, NCT01455402)	107
	**MEDI8897**, MedImmune	**MEDI8897** is an anti-RSV antibody isolated a human B cell with significantly higher potency than palivimab and an Fc-modification to extend half-life. This mAb is in Phase 2 trials (NCT02878330)	108
	**REGN2222**, Regeneron Pharmaceuticals	**REGN2222** is an IgGmAb targeting the RSV-F protein. This mAb is in Phase 3 trials for prevention of RSV in pre-term infants with lower respiratory tract infection (NCT02325791)	155
	**ALX-0171**, Ablynx	**ALX-0171** is in Phase 2 trials for infants hospitalized for respiratory syncytial virus lower respiratory tract infection (RSV LRTI). It is a single-domain camelid-derived antibody, or nanobody	156
Ebola	**ZMapp**, National Institute of Allergy and Infectious Diseases (NIAID)	**ZMapp** is an optimized combination of three antibodies. It was given special approval for compassionate use during the 2014/2015 Ebola outbreak and is in Phase 1 trials (NCT02389192)	113, 115, 157
Rabies	**CL184 (CR57 and CR4098)**, Crucell	**CL184** is a mAb cocktail consisting of **CR57 and CR4098**, and **CL184** was evaluated as a replacement for human rabies immunoglobulin (HRIG) (NCT00708084, NCT00656097, NCT0122838)	123
	**RAB-1 (17C7)**, Serum Institute of India and MassBiologics	This antibody was developed using transgenic HuMab-Mouse (Medarex) and it has been tested in multiple clinical trials in India ((CTRI/2009/091/000465 and CTRI/2012/05/002709)	130, 131

#### Human cytomegalovirus

HCMV is a member of the herpes family. Like other members of this family, HCMV can establish lifelong latent infection in its host, with occasional reactivation.^43^ Infection is in general asymptomatic, but can cause serious disease in people whose immune systems are compromised, such as transplant recipients and AIDS patients with HIV infection. Furthermore, children infected with HCMV in utero are at risk for serious birth defects. They may even experience delayed hearing loss and deafness despite being asymptomatic at birth.^43^ After several decades of effort, an effective vaccine against HCMV infection remains elusive.^44^

Typical adult therapy for HCMV infection is with antivirals such as ganciclovir, foscarnet, and cidofovir.^45^ For children who are suspected to have been infected in utero, treatment with Ganciclovir may prevent developmental problems and loss of hearing.^46^ Women with primary infection during pregnancy, a risk factor for congenital HCMV infection and disease (cHCMV), may be treated with HCMV immunoglobulin as a way to reduce the cHCMV risk.^47^ Polyclonal preparations of antibodies (IVIGs) are an alternative for antiviral medications that have serious limitations. For example, IVIGs are difficult to standardize and less effective. In contrast, mAbs specifically targeting key epitopes should provide an advantage in efficacy. Multiple HCMV targeting mAbs being developed are in various stages of preclinical and clinical trials.^48, 49^

HCMV is a complex virus with multiple antigens, including glycoprotein B (gB) and the gHp entameric complex.^43, 50^ The vast majority of antibodies generated against HCMV target its gB antigen. The gB targeting antibodies alone may not have strong neutralizing ability to control HCMV infection and reactivation.^51^ Recent studies have demonstrated that the pentameric gH complex is the primary target for neutralizing antiviral antibodies,^50^ and as result most recently developed mAbs target the virus’ pentameric complex.^51^ The use of a combination of mAbs has several advantages, including enhanced efficacy and decreased development of viral resistance. At least two of the HCMV targeting antibodies in clinical trials are combinations of two mAbs.^48, 49^

##### CSJ148

CSJ148 is a combination of two anti-HCMV human mAbs -LJP538, which binds to the viral gB protein and LJP539, which binds to the viral gH pentameric complex.^48^ LJP538, also known as 7H3, and LJP539, as 4I22, were isolated from EBV immortalized B cells from HCMV-immune human donors as described.^52, 53^ Results from clinical trials show CSJ148 and its component mAbs were safe and well tolerated, with pharmacokinetics as expected for human immunoglobulin.^48^ Phase 2 clinical trials of CSJ148 in stem cell transplant patients are ongoing.^54^

##### RG7667

RG7667 is a combination of two mAbs, MCMV5322A and MCMV3068A.^49^ MCMV5322A is an affinity-matured version of MSL-109 that binds a neutralizing epitope on HCMVgH/gL.^55^ MSL-109 is a human mAb isolated from spleen cells of a HCMV seropositive individual. In the late 1990s, MSL-109, known by several other names including Protovir, SDZ 89-109, SDZ MSL-109, and Sevirumab, was developed by Sandoz in several clinical trials as a therapy for HCMV infection.^56^ Development was discontinued after MSL-109 failed to demonstrate improved outcomes in the treatment of HCMV retinitis in AIDS patients and prevention of HCMV infection after hematopoietic stem cell transplantation.^56^ In addition to the activities in developing MSL-109 as a therapy, the antibody was a subject for a series of mechanistic studies, including some that defined the mechanism by which HCMV escapes neutralization by MSL-109^57^. The other component of RG7667, MCMV3068A, binds the pentameric gH complex.MCMV3068A was isolated from a mouse hybridoma and subsequently humanized.^49^

RG7667 is developed by Genentech as a treatment to prevent HCMV infection in utero and in solid organ and hematopoietic stem cell transplant recipients. Phase 1 studies of RG7667 showed to be safe and well-tolerated and had a favorable pharmacokinetic and immunogenicity profile. The study supports further development of RG7667 as a therapy for the prevention and treatment of HCMV infection in susceptible populations.^49^ In a Phase 2 trial in high-risk kidney transplant recipients, RG7667 was well tolerated, numerically reduced the incidence of HCMV infection within 12 and 24 weeks post-transplantation; it was statistically significant for delaying time to HCMV viremia, and was associated with few cases of HCMV disease compared to placebo.^58^

#### Influenza

Influenza virus infections are common and usually cause only mild illness. Typical therapy involves respiratory precautions and medication such as oseltamivir (Tamiflu), that inhibit the influenza protein neuraminidase involved in release of virus particles. However, development of resistance to neuraminidase inhibitors is a problem.^59^ Influenza viruses have a unique flexibility which tolerates small errors, resulting in a change of viral structure known as “antigenic drift” that allows escape of the immune response.^60^ The seasonal flu vaccine protects against the influenza viruses that are predicted to be most common during the upcoming season. However, the vaccine is not always effective, due to mismatch of the predicted strains with the actual circulating flu strains. A universal flu vaccine with long-term effectiveness remains elusive.^61^ Occasionally influenza virus makes a major change that preserves its virulence, as in the case of the 2009 pandemic H1N1 strain.^34^ The large antigenic drift renders the seasonal flu vaccine of little efficacy. As a result of the rapid development of antibody isolation and engineering technologies, passive immunization with broadly neutralizing antibodies is becoming an increasingly viable approach to address the immediate health threat of an influenza pandemic while vaccines are being developed.

##### MHAA4549A

MAb MHAA4549A, also known as 39.29, was cloned from a single-human plasmablast cell isolated from an influenza vaccinated donor. MHAA4549A binds a highly conserved epitope on the stalk of influenza A HA and blocks the HA-mediated membrane fusion in the endosome, and is capable of neutralizing all known human influenza A strains.^62^ In two Phase 1 clinical trials, MHAA4549A was safe and well tolerated up to a single intravenous dose of 10,800 mg. The mAb demonstrates linear serum pharmacokinetics consistent with those of a human IgG1 antibody lacking known endogenous targets in humans.^63^ MHAA4549A is currently in Phase 2 clinical trials for the treatment of patients hospitalized with severe influenza A infection.^64, 65^

##### VIS410

VIS410 is an engineered human IgG1mAb, which targets the stem (or stalk) region of influenza A HA and has demonstrated binding to both group 1 and group 2 HAs of influenza A viruses.^66^ Prophylactic administration of VIS410 resulted in the complete protection of mice from developing acute respiratory distress syndrome against lethal influenza A (H7N9) virus challenge.^67^ In a Phase 1 clinical trial, VIS410 was safe and well tolerated and had good relative exposure in both serum and upper respiratory tract. These results support its use as either a single-dose therapeutic or prophylactic for influenza A.^68^ VIS410 is currently in a Phase 2a study designed to assess the safety and tolerability of the antibody in subjects with uncomplicated influenza.^69^

##### CR6261

Isolated from combinatorial display libraries that were constructed from human IgM( + ) memory B cells of seasonal influenza vaccinees, antibody CR6261 neutralizes the virus by blocking conformational rearrangements associated with membrane fusion. The antibody is protective in mice when given before and after lethal H5N1 or H1N1 challenge.^5^ CR6261 recognizes a highly conserved helical region in the membrane-proximal stem of HA1 and HA2. The antibody neutralizes the virus by blocking conformational rearrangements associated with membrane fusion.^70^ CR6261 is currently in Phase 2 clinical testing.^71^

##### CR8020

The human mAb CR8020 has broad neutralizing activity against most group 2 viruses, including H3N2 and H7N7, which cause severe human infection. CR8020 was isolated from a B cell of a donor vaccinated against influenza.^72^ The crystal structure of Fab CR8020 with the 1968 pandemic H3 HA reveals a highly conserved epitope in the HA stalk distinct from the epitope recognized by the V(H)1-69 group 1 antibodies.^72^ CR8020 has been tested in Phase 1/2trials.^73^ Structural and computational analyses indicate that CR8020 targets HA residues that are prone to antigenic drift and host selection pressure. Critically, CR8020 escape mutation was seen in certain H7N9 viruses from recent outbreaks.^74^

##### TCN-032

Antibody TCN-032 was isolated from ab IgG( + ) memory B cell of a healthy human subject. The antibody recognizes a previously unknown conformational epitope within the ectodomain of the influenza matrix 2 protein, M2e.^75^ This antibody-binding region is highly conserved in influenza A viruses. The region is present in nearly all strains detected to date, including highly pathogenic viruses that infect primarily birds and swine and the 2009 swine-origin H1N1 pandemic strain (S-OIV). In addition, TCN-032 protected mice from lethal challenges with either H5N1 or H1N1 influenza viruses.^75^ A Phase 1 clinical study showed that TCN-032 was safe, with no evidence of immune exacerbation based on serum cytokine expression. The trial also showed that the antibody may provide immediate immunity and therapeutic benefit in influenza A infection, with no apparent emergence of resistant virus.^76^

#### Human immunodeficiency virus

Despite decades of intensive effort, an effective HIV vaccine remains a challenge. With recent advances in the identification of broadly neutralizing antibodies from single memory B cells of infected individuals, anti-HIV antibodies are becoming a viable approach for both prophylactic and therapeutic treatment of HIV infection and AIDS.^77^ A number of broadly HIV-1 neutralizing antibodies are currently in clinical development to assess their therapeutic benefit in passive immunization. Most of the HIV mAbs in clinical testing, for instance VRC01, 3BNC117, 10-1074, and 4E10, are broadly neutralizing on the basis of targeting conserved viral epitopes.^78^ PRO 140 is an antibody used to treat HIV targeting host CCR5, which often acts with CD4 as a co-receptor for the virus.^79^ Many excellent reviews provide more detail on broadly neutralizing antibodies against HIV.^77, 80–82^ Here we highlight some examples of HIV targeting antibodies in clinical development.

##### VRC01

VRC01 is a broadly neutralizing HIV-1 mAb isolated from the B cells of an HIV-infected patient.^40, 83^ It is directed against the HIV-1 CD4-binding site and is capable of potently neutralizing diverse HIV-1 strains.^84^ A Phase 1 study showed that the antibody was safe and demonstrated expected half-life and pharmacokinetics for a human IgG.^85^ In two open-label trials of the safety, side-effect profile, pharmacokinetic properties, the antiviral activity of VRC01 was tested in persons with HIV infection who were undergoing ART (antiretroviral therapy) interruption. The antibody slightly delayed plasma viral rebound in the trial participants as compared with historical controls, but did not maintain viral suppression through week 8^86^ VRC01 is being tested in multiple Phase 2 trials.^87–89^

##### 3BNC117

Human antibody 3BNC117was isolated from single B cells of a patient with high titers of broadly neutralizing antibodies. This antibody binds to an HIV gp120 core glycoprotein stabilized in the CD4-bound conformation and lacking the variable (V) loops 1 to 3.^90^ Antibody 3BNC117 blocked infection and suppressed viremia in macaques infected with the R5 tropic simian-human immunodeficiency virus (SHIV)-AD8, which emulates many of the pathogenic and immunogenic properties of HIV-1 during infections of rhesus macaques.^91^ In Phase 1 trial, 3BNC117 is safe and effective in reducing HIV-1 viremia.^92^ In a Phase 2a trial, 3BNC117 suppresses viral rebound in humans during treatment interruption. The antibody exerts strong selective pressure on HIV-1 emerging from latent reservoirs during analytical treatment interruption in humans.^93^ In addition to suppressing viremia in HIV-1-infected individuals, 3BNC117 can enhance host humoral immunity to HIV-1^94^. Antibody 3BNC117 is also being tested in combination with a functionally similar broadly neutralizing antibody, 10–1074, in the treatment of HIV-1 infection.^91, 95, 96^

##### 4E10, 2F5, and 2G12

Antibodies that recognize the highly conserved membrane proximal external region in the gp41 ectodomain stem of HIV such as 4E10, 2F5, and 2G12 have been tested in Phase 1/2 trials in well-suppressed HAART-treated individuals treated during acute and early HIV-1 infection.^97^

##### Pro 140, Ibalizumab, and bispecific antibodies

In addition to antibodies directly targeting viral antigens to prevent and treat HIV-1 infection, antibodies targeting host receptors such as CCR5 and CD4 receptor are also being developed for HIV infection. For example, the CCR5 targeting humanized IgG4 mAb Pro 140 has been tested in clinical trials and the antibody exhibited potent, long-lived antiviral activity and was generally well tolerated.^79, 98, 99^ Ibalizumab (iMab), a humanized mAb that binds to a conformational epitope on CD4 and blocks entry of HIV-1, is also being tested in clinical trials.^100^ In another approach, bispecific Abs that combine the HIV-1 inhibitory activity of ibalizumab with that of anti-gp120 bNAbs were constructed for passive immunization to prevent HIV-1 infection.^101, 102^

#### Respiratory syncytial virus

##### Palivizumab, motavizumab, and motavizumab-YTE

In 1998, the FDA approved palivizumab, which binds to the F glycoprotein of the RSV for prophylaxis in children susceptible to severe disease.^103, 104^ Motavizumab is an affinity matured derivative of palivizumab, tenfold more potent than palivizumab in F glycoprotein binding.^103, 105^ In Phase 3 clinical trials, motavizumab recipients had a 26% relative reduction in RSV hospitalization compared with palivizumab recipients, and motavizumab was superior to palivizumab for reduction of RSV-specific outpatient lower respiratory tract infections (MALRIs, 50% relative reduction).^106^ A half-life extended derivative of the antibody, known as motavizumab-YTE (motavizumab with amino-acid substitutions M252Y/S254T/T256E [YTE]), an Fc-modified anti-RSV mAb was also tested in Phase 1 trials.^107^ Clearance of motavizumab-YTE was significantly lower (71 to 86%) and the half-life was two to fourfold longer than that of motavizumab in healthy participants.^107^ The sustained serum concentrations of motavizumab-YTE were fully functional, as shown by RSV neutralizing activity that persisted for 240 days with motavizumab-YTE versus 90 days postdose for motavizumab.^107^ Despite the improvements of motavizumab and motavizumab-YTE over palivizumab, the clinical benefits were considered incremental and they have not been approved for clinical use.

##### MEDI8897, REGN2222, and ALX-0171

MEDI8897, which is 100-fold more potent than palivizumab in vitro, is derived from D25^108^ a mAb isolated from a B cell of a human donor targeting the perfusion conformation of the RSV F protein. MEDI8897 was engineered with the YTE substitution within its Fc region for extended half-life. In a Phase 1 study, the mean half-life of MEDI8897 was 85 to 117 days across dose groups, and the safety profile of MEDI8897 was similar to placebo.^108^ MEDI8897 is currently in a Phase 2b trial in healthy preterm infants who are between 29 and 35 weeks gestational age and entering their first RSV season.^109^ REGN2222, another human IgG mAb targeting the RSV-F protein is currently under Phase 3 clinical testing.^110^ Not an IgG1 antibody, ALX-0171 is a single-domain camelid-derived antibody, or nanobody, targeting the RSV-F protein. Due to small size of the nanobody, ALX-0171 is being tested as a therapeutic by inhalation in Phase 2 in infants (aged 1–24 months) who were hospitalized with an RSV infection.^111^

#### Ebola, Zika, rabies, and HBV

An outbreak of Ebola in West Africa between 2014 and 2015 affected 28,652 people and led to more than 11,325 deaths.^112^ The lessons learned from the handling of that high mortality rate epidemic contribute to the call for taking innovative counter measures against emerging infectious diseases, including the use of mAbs. For example, an experimental mAb cocktail ZMapp was given special approval for compassionate use during the fast-moving epidemic. ZMapp is a combination of three mAbs (c13C6, c2G4, c4G7) optimized from two previous antibody cocktails (ZMab and MB-0033).^113^ ZMapp showed protective efficacy in a rhesus macaques challenge model.^113^ However, a randomized, controlled human trial of ZMapp plus the current standard of care did not meet the statistical threshold set for improved efficacy as compared with the current standard of care alone (NCT02363322)^114–116^. Efforts to isolate potent neutralizing antibodies against Ebola are ongoing. For example, potent neutralizing antibodies targeting the Ebola virus surface glycoprotein (EBOV GP) were isolated through sequential immunization of rhesus macaques and antigen-specific single B-cell sorting.^117^ Similarly, potent neutralizing antibodies targeting the Ebola EBOV GP were isolated from the peripheral B cells of a convalescent donor who survived the 2014 EBOV Zaire outbreak.^118^ These highly potent neutralizing mAbs could serve as promising candidates for prophylactic and therapeutic interventions against Ebola.

The outbreak of Zika in 2015–2016 and its association with congenital abnormalities also increased awareness of the urgent need to take measures against emerging infectious diseases.^119^ Similar to combating Ebola and other viral infections, development of vaccines is the top priority to protect the population from Zika infection.^120^ Antibody-based therapeutics are also proposed as an option for the treatment of Zika infection.^23^ For example, two antibodies with potent ZIKV-specific neutralization, isolated from a single patient, provided postexposure protection to mice in vivo.^23^ Structural studies revealed that Z23 and Z3L1 bound to tertiary epitopes in envelope protein domain I, II, or III, indicating potential targets for ZIKV-specific therapy.^23^

Rabies occurs worldwide and it is almost invariably fatal once clinical symptoms develop.^121, 122^ Rabies is mostly preventable if post-exposure prophylaxis (PEP) is administered before clinical symptoms develop. PEP consists of thorough wound cleansing followed by immediate administration of rabies immune globulin (RIG), together with a full course of rabies vaccination.^123^ However, due to the lack of education and availability of RIGs and vaccines, it was estimated that global canine rabies causes about 59,000 deaths mostly in Asia and Africa.^121^ As the supply of human RIG (HRIG) and equine RIG (ERIG) is often limited, it is desirable to develop potent neutralizing human mAbs to replace RIGs.^123^ CL184 is mixture of two human mAbs:CR57 and CR4098. Mab CR57, which binds to an epitope of the rabies glycoprotein, was isolated from a EBV-transformed memory B cell from a human subject vaccinated with inactivated rabies virus.^27, 124, 125^ MAbCR4098, which recognizes a non-overlapping epitope of mAbCR57, was isolated from phage-displayed antibody libraries constructed from PBMCs of rabies virus vaccinated human subjects.^126^ CL184 has been evaluated in multiple Phase 1/2 clinical studies to be safe and efficacious.^123, 127–129^ HuMab 17C7, also known as RAB1 which was generated from transgenic mice carrying human immunoglobulin, is another potent rabies virus neutralizing mAb that has been tested in multiple clinical trials in India (CTRI/2009/091/000465 and CTRI/2012/05/002709).^130, 131^ There are continuing efforts to identify more potent and broadly neutralizing mAbs against rabies. For example, two antibodies with potency and broad-spectrum reactivity, RVC20 and RVC58, were identified from immortalized B cells of vaccinated donors.^132^ RVC20 and RVC58 were able to neutralize all 35 strains of rabies virus in vitro. Furthermore, they showed higher potency and breadth compared to antibodies under clinical development, including CR57, CR4098, and RAB1.^132^

Hepatitis B can establish both acute and chronic infections that lead to serious liver disease. Maternal to fetal transmission is one important route keeping prevalence high in regions where the hepatitis B is endemic. Hepatitis B immunoglobulin antibody preparations are one form of countermeasure under investigation.^133^ mAbs are also under development to supplement the suite of tools available to control the spread of HBV and associated disease.^134, 135^

#### Dengue

Dengue is the world’s most prevalent mosquito-borne disease, with an estimated 390 million people each year becoming infected.^136^ Severe dengue, associated with infection with different strains of the virus, is characterized by vascular permeability, bleeding from mucosa and intestinal tract, dengue shock syndrome, and acute renal failure.^137^ In contrast with HCMV where severe symptoms are accompanied by a detectable active phase,^138^ dengue symptoms often appear after the peak of viremia;^139^ therefore, an antibody applied for passive immunotherapy would have to be used before the onset of symptoms to be early enough to avoid viremia. Another difference between HCMV and dengue is that for HCMV, developing fetuses or infants and people with compromised immune systems are clearly at risk for the development of severe disease.^43^ In contrast, there are no clear prognostic indicators for susceptibility to severe dengue virus disease. Such prognostic indicators are necessary due to practical limitations on the number of people who can receive passive immunotherapy. Further, dengue antibodies at sub-neutralizing concentrations may enhance uptake of dengue virus in a way that dramatically aggravates symptoms (antibody-dependent enhancement, ADE).^136^ Therefore, the development of vaccines has been the focus for dengue control. So far one vaccine has been approved, but the vaccine (CYD-TDV, Dengvaxia, Sanofi Pasteur) is less than ideal and better dengue vaccines are urgently needed.^140^

mAbs against dengue have been generated so their interaction with dengue could be analyzed to identify the virus’ best target epitopes, a key first step in vaccine design. A summary of these epitopes and their associated antibodies is presented in several excellent reviews.^136, 141^ The epitopes targeted by antibodies most effective at neutralizing dengue virus were grouped into broad classifications based on whether these epitopes were present in a single virion (epitopes on a monomer) or were composed of features on more than one virion (quaternary epitopes). Epitopes on a monomer were further grouped according by region of the dengue virion bound: prM protein, FLE (amino acids 98–113), BC loop E protein domain II (amino acids 73,78, and 79), or EDIII. Quaternary epitopes were also sub-divided into EDE epitopes (EDI, EDII, and EDIII); E protein epitopes (monomer EDI, EDI-EDII hinge, and EDIII; intact virion only); and E protein herring-bone epitope (EDI, EDI-EDII hinge, and EDIII). Although no dengue antibody therapeutics have reached the stage of clinical trials, some of broadly neutralizing antibodies have the potential to be developed into therapeutics against dengue infections.^142–148^

Dengue targeting antibodies at high concentration may enhance infection. Therefore, therapeutic antibodies need to be engineered to abolish the interaction of antibodies to the FcγRs on macrophages, thus preventing ADE.^149^ For example, a N297A mutation in the Fc region of D23-1G7C2 IgG1 was engineered to reduce the affinity of the IgG1 Fc region for FcγRs, resulting in a marked reduction in ADE activity in in vitro cell studies.^150^ In another study, dengue neutralizing mAbs targeting distinct epitopes on the four DENV serotypes were engineered to prevent FcγR binding by introducing LALA (L234A-L235A) mutations in the IgG1 Fc region. The LALA variant did not enhance infection and neutralized DENV in vitro and in vivo as postexposure therapy in a mouse model of lethal DENV infection.^151^

### Perspective

Only one mAb, specific to RSV, has been approved for prophylaxis use of viral infections. Several challenges impede the progress of the many antiviral mAbs in the pipeline. One key challenge is the relatively small market for antibody treatments of viral diseases, and potentially higher cost associated with production of recombinant antibodies, as compared to small molecule antivirals.

Another key challenge is the competition of other forms of treatment and prevention. Vaccines are often still the best approach to control viral infections, often with benefits of life-long immunity. Even after effective mAb therapies are developed, their widespread application may be impractical due to the high costs of conventional production compared to other countermeasures against disease from viral infections. One way to address the problem of the high cost of mAb therapies is to reduce the cost of production. For instance, production of mAbs in scFv or Fab form or as camelid nanobodies enables relatively inexpensive expression in prokaryotic systems.^152^ Another way to address the problem of high costs is financing through partnerships with traditional donors and newer funding sources such as the Gates Foundation. For example, development impact bonds have been tested as a way to eliminate rabies infection in dogs, in turn reducing demand for expensive treatments for humans.^153^

A third key challenge is the complexity of pathology, epidemiology, and immunology that can be associated with infection. The way kinetics of infection informs therapeutic strategy, for instance, explains why no dengue mAbs are in clinical trials and why vaccination is the preferred method to control influenza. For dengue and influenza A, symptoms often appear after the peak of viremia;^139, 154^ an antibody applied for passive immunotherapy would have to be used before the onset of symptoms to be early enough to avoid viremia. One potential solution is matching a therapeutic antibody with a rapid, point of care diagnostic test. The diagnostic could be used to identify patients with infection and susceptibility to severe disease who would benefit from passive immunotherapy with the therapeutic antibody. Finally, viruses can have complexity that prevents development of a single lasting treatment, for instance multiple strains, rapid evolution, and obscure mechanisms of infection and neutralization escape.

As discussed above, Fc region engineering has been shown effective for increasing the half-life of therapeutic antibodies, for instance the RSV mAb motavizumab YTE, and for preventing ADE through reduced FcR binding, as for dengue antibodies. The interactions of the Fc domain with diverse other receptors provides additional opportunities for engineering to optimize therapeutic efficacy of antibodies. The advantages and disadvantages of engineering approaches was recently reviewed by Bournazos and Ravetch.^155^

Passive transfer of antibodies for the prevention and treatment of viral infections is easy to manage, but the high cost associated with antibody therapies is prohibitive in prophylactic use of antibodies for intractable viruses such as HIV and influenza A virus for a large population, particularly in low-resource areas.^156^ One alternative to passive administration of antibody therapies involves the delivery of transgenes encoding well characterized neutralizing antibodies by a vector. The transgenes direct the expression of antibodies in non-hematopoietic cells, which then secrete mAbs into circulation. It has been demonstrated that intramuscular delivery by electroporation of synthetic DNA plasmids engineered to express modified human mAbs against multiple DENV serotypes confers protection against DENV disease and prevents ADE of disease in mice.^157^ More work is needed before clinical applications, but vector delivery of prophylactic or therapeutic antibodies against viral infections is a promising alternative to the current practice of passive transfer.
